# Impact of Simulated Vascular Aging and Heart Rate on Myocardial Efficiency: A Tale of Two Paradigms from In Silico Modelling

**DOI:** 10.3390/jcdd12050163

**Published:** 2025-04-22

**Authors:** Lawrence J. Mulligan, Julian Thrash, Ludmil Mitrev, Daniel Ewert, Jeffrey C. Hill

**Affiliations:** 1 Department of Anesthesiology, Cooper University Hospital, Camden, NJ 08103, USA; mitrev-ludmil@cooperhealth.edu; 2 Cooper Medical School, Rowan University, Camden, NJ 08103, USA; 3 Department of Biomedical Engineering, University of North Dakota, Grand Forks, ND 58202, USA; julian.thrash@und.edu (J.T.); dan.ewert@und.edu (D.E.); 4 School of Medical Imaging and Therapeutics, Massachusetts College of Pharmacy and Health Sciences University, Worcester, MA 02115, USA; jeffrey.hill@mcphs.edu

**Keywords:** aortic compliance, pressure–volume area, myocardial oxygen consumption, myocardial efficiency, mechanical efficiency, computational modeling

## Abstract

Introduction: Vascular aging is associated with a loss of aortic compliance (C_A_), which results in increased left ventricular pressure–volume area (PVA), stroke work (SW) and myocardial oxygen consumption (MVO_2_). Myocardial efficiency (MyoEff) is derived from the PVA and MVO_2_ construct, which includes potential energy (PE). However, the SW/MVO_2_ ratio does not include PE and provides a more accurate physiologic measure. Methods: We used a modified computational model (CM) to assess PVA and SW and calculate MVO_2_ using a pressure-work index (e MVO_2_), to derive MyoEff–PVA and MyoEff–SW metrics. Phase I evaluated five levels of human C_A_ from normal (N) to stiff (S) at 80 bpm, and Phase II evaluated two levels of C_A_ (N and S) at three heart rates (60, 100, and 140 bpm). Results: During Phase I, MyoEff–PVA increased from 20.7 to 31.2%, and MyoEff–SW increased from 14.8 to 18.9%. In Phase II, during the N setting coupled with increases in the heart rate, the MyoEff–PVA decreased from 29.4 to 14.8 to 9.5%; the MyoEff–SW also decreased from 22.5 to 10.3 to 5.9%. As expected, during the S setting, MyoEff–PVA decreased from 45.5 to 22.9 to 14.8; a similar effect occurred with the MyoEff–SW, demonstrating a decrease from 29.9 to 13.9 to 7.9%, respectively. Conclusions: The CM provided insights into a simple and clinically relevant calculation for assessing MyoEff. The agreement on the CM metrics aligns with studies conducted previously in the clinical setting.

## 1. Introduction

Insights regarding cardiac work and energetic metrics from experimental and clinical studies were developed from the seminal work by Suga and colleagues quantifying the left ventricular pressure–volume (PV) construct [1,2,3,4,5,6,7]. The PV construct allows for comparing mechanical efficiency (ME) and myocardial efficiency (MyoEff) [5,6]. Mechanical efficiency is derived from stroke work (SW) divided by the pressure–volume area (PVA) and has been shown to be altered in various physiologic and disease states [8,9,10]. Mechanical efficiency (SW/PVA) is an engineering perspective and is intended to be a complementary metric to the PV indices of contractility.

The ME and MyoEff metrics have been studied in chronically instrumented canine models and patient studies [9,11,12], and these metrics are not interchangeable [8,9]. The MyoEff metric can be derived using either the SW or the PVA variables along with myocardial oxygen consumption (MVO_2_). The SW/MVO_2_ metric (MyoEff–SW) yields a lower value than the PVA/MVO_2_ (MyoEff–PVA) metric but represents the metabolic efficiency related to completed work [9,13]. Several studies have used the MyoEff–SW metric and quantified how disease states impact this measurement [8,9,10].

Assessing MVO_2_ has evolved from invasive measures to the development of the pressure-work index (PWI) by Rooke and Feigl [14] and noninvasive measures using positron emission tomography (PET) imaging [15]. Combining PET with transthoracic echocardiography offers a practical tool to measure the SW, MVO_2_, and MyoEff–SW metrics. However, the ability for the wide use of PET for serial evaluation is low. The PWI or estimated MVO_2_ (eMVO_2_) were highly correlated with the PET measures of MVO_2_ [15]. Combining echocardiography measurements of SW and left ventricular mass with arterial pressures to estimate MVO_2_ offers a noninvasive method for the serial evaluation of MyoEff. Other conventional, indirect measures of MyoEff, such as the rate pressure product and mechano-energetic efficiency, offer insights but do not provide a true ratio [16]. A comparison of indirect measures has been studied, and while the PVA tool was determined to be the best metric, its invasive nature reduces its clinical utility.

Over the past three decades, numerous publications have revisited the importance of aortic compliance (C_A_) in cardiovascular health [17,18,19]. Aortic compliance refers to the ability of the aorta to expand and contract in response to changes in blood volume. These findings have consistently shown that a loss in C_A_ is associated with an elevation of central aortic blood pressure, decreases in cardiac function, and an impact on SW [20]. The impact of simulated VA on eMVO_2_ and MyoEff remains to be evaluated.

Due to the previously described methods using the PVA construct and the inability to conduct invasive serial studies, we used a computational model (CM) to investigate how vascular aging impacted the metrics quantifying cardiac function and ventricular vascular coupling (VVC). The CM generated PV loop parameters along with eMVO_2_ in a simulated aging environment as C_A_ was reduced from “normal to stiff”. We coupled this with the impact of an elevated heart rate, providing insights into the two paradigms: MyoEff–PVA and MyoEff–SW.

## 2. Methods

### 2.1. Computational Model Concept

**Figure 1:**
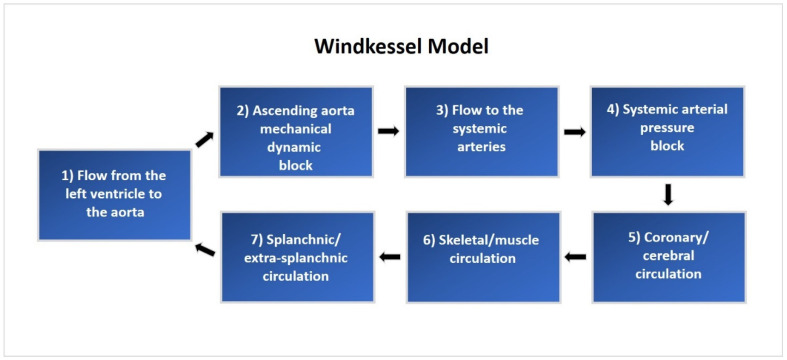
Flow schematic representing the human cardiopulmonary system with the Windkessel model.

A lumped parameter CM was implemented using MATLAB (2020b release, MathWorks, 1 Apple Hill Drive, Natick, MA, USA). This model includes 240 mathematical equations based on a comprehensive representation of the human cardiopulmonary system [20,21,22]. The CM is composed of a five-compartment systemic vascular system, pulmonary circulation, lung mechanics, and blood gas exchange, as well as the left and right atria and ventricles. Hemodynamic function is regulated by autonomic neurological control and local autoregulation based on blood gas concentrations. These integrated mathematical elements work together to create an accurate model of the human cardiopulmonary system. A system overview of cardiovascular circulation through the proximal and distal Windkessel model used in this study is illustrated in Figure 1.

### 2.2. Baseline Model Verification

**Figure 2:**
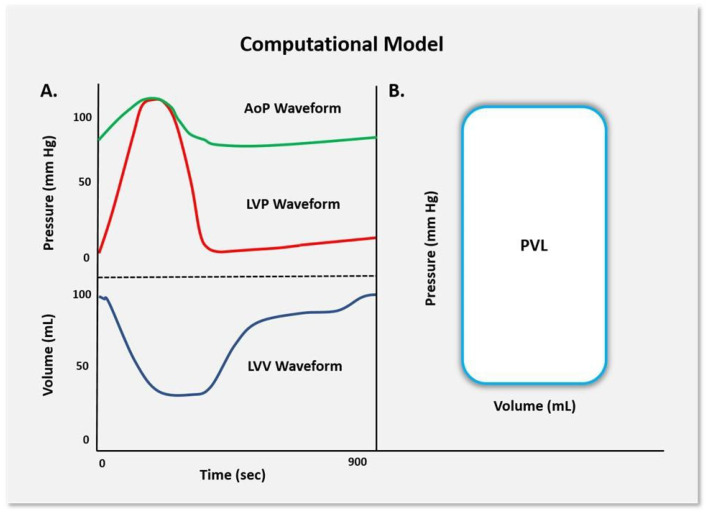
(**A**) Schematic signals generated by the CM include the LV pressure and aortic pressure waveforms (above) with the simultaneous LV volume signal (below). (**B**) The combined LV pressure and volume construct was generated.

The integrity of the CM was assessed by setting the C_A_ and vascular peripheral resistance parameters within the model to their published values and evaluating the simulated cardiovascular response based on published PV study data [8,9]. Nominal C_A_ and vascular peripheral resistance (R_T_) were changed to 0.7 mL/mmHg and 1.28 mmHg·mL^−1^·s^−1^, respectively, based on prior studies of normal cardiovascular physiology [23,24]. We directly compared the cardiovascular waveforms generated by the model with key measurements, including the left ventricular pressure, left ventricular volume, aortic pressure, and the PV loop response (Figure 2).

Model verification was based on the agreement of simulated PV data versus established normal physiological cardiovascular responses. This process involved fine-tuning the ancillary system parameters of the CM to match the original model’s waveforms and standard human physiological cardiac PV responses. This alignment with typical human physiology assures the model’s accuracy. Under normal compliance and resistance conditions, the model produced PV waveforms that resulted in a systolic pressure of approximately 120 mmHg, a diastolic pressure of 80 mmHg, and a pulse pressure of 40 mmHg.

### 2.3. Experiment Setup

After verifying the model’s capability to recreate normal physiologic cardiovascular responses, it was adapted to fit the experimental setup and procedure of the canine experiment described by Kelly et al. [25]. The model was modified to recreate the scenario used in invasive PVL studies, which includes right atrial pacing, autonomic nervous system blockade, and a decrease in preload through vena cava occlusion (VCO). The nominal vascular compliance and peripheral resistances were adjusted to represent normal and stiff conditions, mimicking human cardiac and arterial function and physiology. The developed model illustrates venous blood flow to the right atrium through the superior and inferior vena cava, representing the total summation of flow converging through the veins in the thoracic cavity. This flow convergence is modeled by pooling the total venous blood flow from the five vascular compartments.

### 2.4. Vena Caval Occlusion

Occlusion of the venous return was simulated using a time-based increase in the hemodynamic resistance of the thoracic cavity veins after allowing the system to reach a steady state three minutes before the VCO. The occlusion occurred proximal to the tricuspid valve and downstream of the five systemic vein sub-compartments. This in silico approach simulates venous return occlusion through a balloon-expandable catheter delivery system, which mimics a reduction in the venous blood return.

### 2.5. Simulated Vascular Aging

Validation of the CM involved the assessment of its response and capability to simulate the effects of progressive vascular aging on cardiac function and efficiency. Vascular aging of the aorta and systemic vascular resistance described in the Kelly et al. study [25] was simulated within the CM by the direct modification of the proximal C_A_ and peripheral vascular resistance elements. Due to the variance in vascular compliance and resistance between a human arterial system and a canine subject, and additional verification between the human CM cardiovascular response and the canine pressure–volume study results was performed to determine parity. A comparable response was found between the native arterial parameters outlined in the Kelly et al. study [25] (C_A_ of 1.65 mL/mmHg and R_T_ of 3.04 mmHg·mL^−1^·s^−1^) and the nominal CM arterial parameters (C_A_ of 0.7 mL/mmHg and R_T_ of 1.28 mmHg·mL^−1^·s^−1^). The stiff Tygon tubing parameters (C_A_ of 0.19 mL/mmHg and R_T_ of 3.66 mmHg·mL^−1^·s^−1^) created a similar cardiac function and efficiency response when set in the CM. The progression of vascular aging was simulated by proportional scaling of the arterial compliances and resistances of the five compartments and aorta starting from nominal settings and linearly scaling compliance to stiff Tygon conditions. The creation of the variables including the stroke volume, aortic flow, and left ventricular pressures were derived from the Windkessel properties of the model. Further details on this process have been published in our prior study [20].

The CM was evaluated during two phases. In Phase I, right atrial pacing was simulated at 80 bpm across the five levels of compliance during each VCO simulation. In Phase II, the normal and the stiff C_A_ settings were evaluated at 60, 100, and 140 bpm. Beat-to-beat data were collected by simulating the VCO under all levels of compliance. The results were verified through an examination of end-diastolic volume (EDV), end-systolic volume (ESV), end-systolic pressure (Pes), end-diastolic pressure (EDP), PP, dP/dt_max_, and PVA.

### 2.6. Evaluation of Cardiac Function

Similar to clinical and pre-clinical studies, LV volume was calculated for each beat during the simulated VCO. The PVA was described by Suga [1] and the parameters were calculated for each beat during the VCO. The ESPVR relation (Pes and Ves) was fit using a linear least-square algorithm to the following equation [2,3]:

Ees = Pes/(Ees − Vo)

where Ees is the slope of the relation and Vo is its volume-axis intercept [3].

The slope and volume axis of the SW–EDV relation were determined using a linear least squares algorithm and were fit to the following equation [26]:

SW = M_W_ (EDV − V_W_)

where Mw is the slope of the relation and Vw is its volume axis.

The slope and volume axis of the dP/dt_max_–EDV relation were determined in the same beats as used for the ESPVR and pre-load recruitable stroke work (PRSW) relations using a linear least squares algorithm and were fit to the following equation [27]:

dP/dt_max_ = dE/dt_max_/(VED − Vo)

In the slope of the dP/dt_max_–EDV relation, dE/dt_max_ represents the maximum rate of change of LV elastance [28]. In a similar manner, the relation between PVA and EDV was fit to the following equation [29]:

PVA = MPVA × (EDV − VPVA)

where MPVA is the slope of the relation and VPVA its volume intercept.

The calculation of eMVO_2_ is determined with the equation below:

eMVO _2_= K1 × (SBP × HR) + K2 × ([(0.8 × SBP + 0.2 × DBP) × HR × SV] ÷ body weight) + 1.43

where:

SBP = systolic blood pressure (peak systolic pressure, mmHg);
DBP = diastolic blood pressure (mmHg);
HR = heart rate (beats/min);
SV = stroke volume (mL);
BW = body weight (kg) (set to 75 kg);
K1 = 4.08 × 10^−4^;
K2 = 3.25 × 10^−4^.

**Figure 3:**
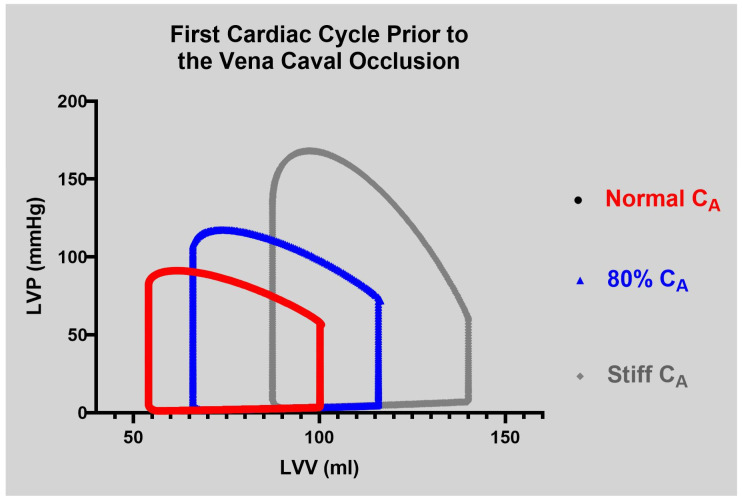
Three pressure–volume loops representing the first cardiac cycle prior to the vena caval occlusion (VCO). Normal, 80% C_A_, and stiff C_A_ loops are presented.

Myocardial efficiency was calculated using two approaches. First, the SW was divided by eMVO_2_, as described by Rooke and Feigl [14]. We chose a left ventricular mass of 200 g in the eMVO_2_ equation, representing a person of 80 kg to produce the steady state MyoEff–SW. In unpublished data, we measured the LV mass in eight subjects with a range of left ventricular mass (87.3 to 217.4 g) and a range of eMVO_2_ (6.1 to 12.5 mL/min/200 g). A comparison of MyoEff–SW versus MyoEff–PVA at a steady state and during the VCO allowed a direct comparison of these metrics. The CM provided five levels of static C_A_ as described above. In both Phase I and II, the parameters were calculated during the first beat of the VCO (steady state, Figure 3) and the transient VCO process.

Phase I and II results were verified and validated through an examination of EDV, ESV, ESP, end-diastolic pressure (EDP), dP/dt_max_, SW, PVA, ESPVR, and eMVO_2_ beat-to-beat data. Validation of the human CM results was conducted through a comparison of the hemodynamic relationships seen in the works of Kelly et al. [25] and Moulton and Secomb [30], and physiological knowledge of the effects of stiffening vascular beds on the heart. Statistical analysis of the data was conducted using GraphPad Prism software Version 10.4 (225 Franklin St, Boston, MA, USA).

## 3. Results

### 3.1. Phase I: Impact of Alterations in Compliance on Ventricular Function, Contractile State, and Myocardial Efficiency

Steady-State Hemodynamics

As expected, the CM generated a simulated loss in C_A_ and the resulting PV loop parameters. The impact on cardiac parameters during steady-state evaluation at 80 bpm with percent changes from normal to stiff are shown in Table 1. The data have been described in detail in a previous publication [20]. Briefly, the impact of the change in C_A_ on the first beat of the VCO run on pressure and volume is pronounced, as shown in Figure 3. As C_A_ was decreased from normal to stiff, several hemodynamic changes occurred. The EDV, ESV, and SV all increased gradually. Additional changes included changes to the dP/dt_max_, which rose by 26.9%, SW, which increased by 90.9%, and PE, which increased by 209.9%.

Several indices of contractile function derived from the PV loop analysis showed notable changes, as presented in Table 2. The EDV–PVA relation increased by 68.6% while the ESPVR decreased by 22.6%. The dP/dt_max_–EDV relation remained relatively flat until the stiff setting, where there was a decrease of 16.6%. The PRSW increased steadily to 32.8%. The C_A_ setting led to a loss of 15.3% in ME from 72.0 to 60.8%. The impact of a loss in C_A_ from normal to stiff at 80 bpm led to an increase of 49.4% in eMVO_2_ from 10.7 to 15.9 mL/min/200 g. The 28% increase in MyoEff–SW was smaller than the change MyoEff–PVA of 50.3% (Table 3).

### 3.2. Phase II: Impact of Alterations in Compliance and Heart Rate on Ventricular Function, Contractile State, and Myocardial Efficiency

After evaluating the physiological response of cardiac function parameters in relation to varying C_A_ conditions and noting the similarities with pre-clinical data and recent findings by Moulton and Secomb [30], we designed the Phase II component to expand upon Phase I. Phase II specifically examined how changes in heart rate (the force-frequency effect) influenced measures of cardiac function, as well as mechanical and myocardial efficiency. The impact of increases in heart rate from 60 to 100 and 140 bpm at the normal and stiff C_A_ is shown in Table 4. From a heart rate of 60 bpm, in both C_A_ settings, as the heart rate increased to 140 bpm, the EDV decreased by 33.4% from 113.2 mL in the normal setting and 32.0% in the stiff setting. Similarly, the ESV decreased by 11.1% and 18.7% at the normal and stiff settings, resulting in large decreases in SV at both settings, of 54.4% and 50.6%. 

Compared to the Phase I data, several changes were observed in the cardiac function metrics. At the normal setting, the ESPVR decreased by 13.2%, the PRSW decreased by 30.7%, and the EDV–PVA decreased by 21.6%. The dP/dt_max_–EDV relation showed an increase of 87.5%. By contrast, at the stiff setting, the ESPVR increased by 29.9% and the dP/dt_max_–EDV increased by 39.8%, while the PRSW and the EDV–PVA decreased by 39.5% and 28%, respectively. Furthermore, the increase in HR resulted in a loss of ME at both settings, with reductions of 18.1% and 19.1%, respectively (see Table 4).

The effects of HR and VA on myocardial energetics were physiologic during Phase II (see Table 5). We observed that increases in HR, along with a reduction in C_A_, were associated with a significant increase in eMVO_2_ of 43.7% (from 9.3 to 13.3).

Additionally, there was a notable decrease in myocardial efficiency across heart rates at the normal and stiff settings (Table 6) measured by MyoEff–SW, which dropped by 73.6% (from 22.5% to 5.9%). A similar decline was seen in MyoEff–PVA, which decreased by 67.6% (from 29.9% to 7.9). In the context of stiff C_A_, we observed an increase in eMVO_2_ of 47.4% (from 14.2 to 20.9). MyoEff–SW decreased by 73.8% (from 29.9% to 7.9%), and MyoEff–PVA showed a reduction of 67.6% (from 45.5% to 14.8%).

## 4. Discussion

### 4.1. Model Rationale

The model was modified to evaluate four key questions: (1) Does the model simulate the effects of VA and increases in heart rate on cardiac function using the pressure–volume construct? (2) Does the addition of the Rooke–Feigl pressure-work index provide an accurate quantification of eMVO_2_? (3) Does the model offer physiological insights into how VA and heart rate influence eMVO_2_ and ME? Finally, (4) how does MyoEff–SW compare with MyoEff–PVA? This model provides an initial perspective on how VA affects cardiac function and energetics in a normal ventricle. All four objectives were successfully achieved, and the model produced PV loop data that align with findings from previous studies. Additionally, the model allows for a direct comparison between MyoEff–SW and MyoEff–PVA.

First and foremost, the CM effectively produced a physiological response in the parameters commonly used to assess both ventricular function and VVC during vascular aging, as previously demonstrated [25,29,31]. Additional studies have underscored the significant impact that an increased afterload has on typical PV loop parameters, which agrees with prior work [20,30]. While earlier research has illustrated how various cardiovascular diseases, such as heart failure and hypertension, increase eMVO_2_ [8,9,10], it is not practical to study this metric serially, which prompted the innovation of the CM. The results of this CM provide compelling evidence that VA is associated with an increase in eMVO_2_.

### 4.2. Evaluation of Contractile Function

#### 4.2.1. Phase I

Previous work that included three experimental animal models evaluated the impact of changes in C_A_ on measures of left ventricular contractility. In the isolated canine model [31], open-chest dog [25], and chronically instrumented model [29], the impact of the loss in C_A_ did not impact ESPVR or PRSW. However, the EDV–PVA change was dependent on the directional change in compliance [29,31]. The difference in these observations is likely due to differences in the animal model and methods used to alter compliance. The CM data agree with several findings from each of these studies, as expected when comparing in vivo to a CM.

The impact of an incremental loss of C_A_ was associated with large increases in EDV, ESV, SW, and end-systolic pressure (Table 1). The increase in EDV is likely to have led to the increase in dP/dt_max_, from 1621 to 2057 (27%) (Table 1). The loss in C_A_ was associated with a moderate loss (22.6%) in ESPVR, a physiologic increase (32.8%) in PRSW, and no change in dP/dt_max_–EDV until the stiff C_A_ setting (a 16.6% loss). These responses are anticipated, as they provide different insights regarding the cardiac contractile state as shown previously [28]. The increase in PRSW is expected, as the loss in C_A_ resulted in an increase in EDV (39.3%) and a large increase in end-systolic pressure (63.6%) (Table 1). The impact of the large increase in EDV also led to a large increase in PVA, with the EDV–PVA relationship increasing by 68% (Table 2). The loss in C_A_ was associated with a 15.3% loss in ME. Similarly, Freeman observed a decrease in ME from 52.5 to 42.5% due to the increase in afterload (similar to a loss in C_A_) [29]. By contrast, Kolh et al. observed no loss in ME, at approximately 71% at both settings of C_A_ [31].

#### 4.2.2. Phase II

In Phase II (Table 4), as the heart rate increased during the normal C_A_ setting, large decreases in EDV, ESV, and SW were observed. Several studies have shown how an increase in HR leads to changes in pre- and afterload with mixed changes in ESPVR. PRSW, dP/dt_max_–EDV, and EDV–PVA [32,33]. The EDV–PVA has been an underutilized metric. The loss of C_A_ leads to a large increase in potential energy (PE), impacting this metric. We observed a large increase in PE as C_A_ decreased (Table 1). The metrics moved in a physiologic manner as HR increased and agreed with the Phase I data at 80 bpm.

During the increase in HR, ESPVR decreased by 12.3% at 100 bpm and 13.2% at 140 bpm compared to 60 bpm. We would have expected a small increase, but the impact of the loss in C_A_ with the increase in HR has not been evaluated previously. In the studies by Freeman and Kelly et al., the impact of an increase in HR at normal and stiff settings were not evaluated [25,29]. The PRSW metric decreased during the normal C_A_ setting (31%) and continued to decrease at the stiff C_A_ setting (39%) as HR increased. Conversely, dP/dt_max_–EDV increased during Phase II with a blunted response at the stiff C_A_ compared to the normal setting. The increase during the normal C_A_ setting of nearly 90% was blunted during the stiff C_A_ setting, resulting in only a 40% increase. The impact of VA and increases in HR on these parameters demonstrates that they contribute uniquely to the change ventricular contractility during changes in afterload and heart rate. The EDV–PVA metric has not been a prominent tool in the literature but does add a valuable tool to understand how the afterload and heart rate impact the relationship between total cardiac work and diastolic volume. As ventricular end-diastolic volumes increased with heart failure and hypertension, the impact of increases in HR led to similar decreases (21% vs. 28%) at both C_A_ settings.

## 5. Ventricular Energetics

### 5.1. The PV Loop Approach

Early work that focused on the assessment of MVO_2_ in subjects presenting with coronary artery disease [34,35] as well as those with left ventricular hypertrophy [36] provided the foundation for future studies using the PV loop construct [8,9,10]. These studies confirmed several physiologic findings, including how an increase in HR impacts the variables that contribute to the calculation of MyoEff–SW. Kameyama et al. studied subjects with different types of cardiac dysfunction with normal and reduced EF [8]. Conventional wisdom dictates that normal EF is associated with normal MVO_2_ and MyoEff–SW. However, in all patients receiving phenylephrine to increase the afterload, MVO_2_ and SW increased, resulting in no change in MyoEff–SW. By contrast, PE increased more than two-fold, leading to a significant increase in MyoEff–PVA [8]. In our CM, PE also increased out of proportion with the change in SW in both Phase I and II (Table 1 and Table 4).

Under the same parameters, Ishihara et al. studied subjects with heart failure and reduced EF at baseline and following dobutamine infusion and found that MVO_2_ and SW increased significantly [10]. Myocardial oxygen consumption demonstrated a greater increase than SW, leading to a loss in MyoEff–SW. This finding suggests that there is an oxygen-wasting effect in this population. The increase in HR in the CM provided similar findings that would be found with inotropic and chronotropic responses. By contrast, Eichhorn et al. studied the impact of beta-blocker therapy in patients with nonischemic dilated cardiomyopathy [37]. The results demonstrated that this therapy reduced MVO_2_ while SW increased, leading to an improvement in MyoEff–SW.

### 5.2. Nuclear-Based Evaluation of MyoEff–SW

Positron Emission Tomography (PET) imaging has allowed the noninvasive assessment of MVO_2_. The work by Vanoverschelde et al. provided several important observations regarding the utility of the Rooke and Fiegl PWI metric [14,15].

A strong correlation (r = 0.92) was found between MVO_2_ and eMVO_2_. In addition, the MyoEff–SW value of 35.0 ± 6.0% was reduced to 30.0 ± 4.0% during dobutamine infusion. The evidence that the PET-derived MyoEff–SW was well-aligned with the Rooke and Feigl method allows for consideration of this approach.

### 5.3. Noninvasive Evaluation of Myocardial Efficiency

Development of the CM was initiated to investigate how vascular aging and HR impacts SW, eMVO_2_, and MyoEff. The CM was utilized to evaluate several iterations to test how VA and increases in heart rate impacted the respective variables. Providing evidence that supports the SW/eMVO_2_ metric, collected noninvasively, this tool represents an opportunity for evaluating MyoEff in patients without the need for imaging or invasive tools. Our results are supported by the previously reported studies listed. Studies conducted by the Framingham group provided clinical evidence that vascular aging impacted pressures, arterial wave reflections, and cardiac function, but those studies did not include a focus on MVO_2_ and MyoEff–SW [36,38,39,40,41]. The current effort was designed to reignite the thought process on these metrics, by creating a simplified, in silico approach.

Previous efforts over the past fifty years focused on the rate-pressure product, the triple rate, and the mechanic-energetic efficiency (MEEi) product [16,32,33]. Each metric can provide directional insights, but they have not been compared against the MyoEff–SW metric. The MyoEff–SW is a direct measure compared to indirect measures.

## 6. Limitations

The current model evaluates how VA alters cardiac function, including metrics of VVC and myocardial energetics in the normal heart. Quantifying changes in complex ventricular and vascular physiology would require employing both finite element and lumped parameter approaches. The CM attempted to replicate pre-clinical animal studies and found agreement in several aspects. However, pre-clinical studies to mimic the impact of VA coupled with elevated heart rates have not been conducted, for obvious reasons. The model did not include parameters to alter the impact of wave reflection or the timing or magnitude on VVC, as that would have necessitated a finite element component. The ventricular component was based on the time-varying elastance construct for the normal heart [42].

The PV loop construct offers valuable insights into cardiac function in both pre-clinical and clinical studies. With the addition of the MVO_2_ measurement, the construct was expanded to include an evaluation of MyoEff, utilizing the PVA-MVO_2_ metric. Several publications have compared SW–MVO_2_ with PVA–MVO_2_ and have demonstrated the that the addition of PE leads to an overestimation of MyoEff [8,9,10]. Moreover, the PVA-MVO_2_ metric has limitations. It relies on invasive measurements, making it unsuitable for serial monitoring. Additionally, it necessitates altering the afterload to simulate a VCO, which poses further challenges in the clinical setting. Furthermore, the PVA–MVO_2_

MyoEff metric is overestimated due to the inclusion of PE in its calculation. By contrast, the MyoEff–SW does not require invasive assessment or load manipulation and provides a clear physiologic measure of MyoEff.

## 7. Conclusions

The development of the CM provided novel insights regarding the impact of simulated vascular aging on metrics of VVC and myocardial efficiency. This in silico approach also provided insights into changes in the LV contractile state, ME, and MyoEff across vascular aging with increased heart rates. The data generated by the CM cannot be conducted in the clinical laboratory but provide compelling evidence on how vascular aging impacts myocardial energetics.

## Figures and Tables

**Table 1:** Impact of loss in C_A_ on cardiac parameters during steady-state evaluation at 80 bpm with percent changes from normal to stiff.

Aortic Compliance	Normal C_A_	90%	80%	60%	Stiff
EDV (mL)	100.6	10.3	16.0	24.0	39.3
ESV (mL)	54.1	14.3	21.9	32.1	61.7
SV (mL)	46.5	5.6	9.2	14.6	13.3
Pes (mmHg)	81.9	19.7	29.1	39.1	63.6
dP/dt_max_ (mmHg/s)	1621	14.7	21.3	27.9	26.9
SW (mmHg∙mL)	3830	25.4	39.9	60.6	90.9
PE (mmHg∙mL)	1523	44.6	70.2	103.9	209.9

Abbreviations: C_A_, aortic compliance; EDV, end-diastolic volume; ESV, end-systolic volume; SV, stroke volume; Pes, end-systolic pressure; dP/dt_max_, rate of rise of left ventricular pressure with respect to time; SW, stroke work; PE, potential energy.

**Table 2:** Impact of loss in C_A_ on parameters of contractile state.

Aortic Compliance	Normal C_A_	90%	80%	60%	Stiff
ESPVR	1.86	−3.8	−7.0	−11.3	−22.6
dP/dt_max_–EDV	16.9	1.8	1.2	−1.2	−16.6
PRSW	71.4	14.3	20.7	28.0	32.8
EDV–PVA	91.7	18.1	27.7	40.1	68.6

Abbreviations: ESPVR, end-systolic pressure-volume; PRSW, preload-recruitable SW; and EDV–PVA, pressure–volume area–end-diastolic volume.

**Table 3:** Impact of loss in C_A_ on metabolic and mechanical efficiency at 80 bpm with percent changes from normal to stiff.

Aortic Compliance	Normal C_A_	90%	80%	60%	Stiff
eMVO_2_	10.7	16.0	24.0	33.0	49.4
MyoEff–SW	14.8	8.1	12.8	20.8	27.8
MyoEff–PVA	20.7	12.8	19.7	30.0	50.3
ME (%)	72.0	−4.2	−6.9	−8.3	−15.3

Abbreviations: eMVO_2_, myocardial oxygen consumption; MyoEff–SW, myocardial efficiency–SW; MyoEff–PVA, myocardial efficiency–PVA; ME, mechanical efficiency.

**Table 4:** Impact of increases in heart rate and decreases in C_A_ from the first cardiac cycle pre-vena cava occlusion.

Aortic Compliance	Normal			Stiff		
HR	60	100	140	60	100	140
		% Change from 60 bpm			% Change from 60 bpm	
EDV (mL)	113.2	−23.2	−33.4	159.6	−22.8	−32.0
ESV (mL)	54.7	−9.4	−11.1	93.0	−15.8	−18.7
SV (mL)	58.5	−36.2	−54.3	66.6	−32.7	−50.6
Pes(mmHg)	82.9	−11.5	−12.9	141.1	−15.4	−15.3
PP (mmHg)	36.6	−29.9	−45.2	115.8	−25.5	−38.5
dP/dt_max_ (mmHg/s)	1086	−7.7	−3.3	1357	−7.1	−3.4
SW (mmHg∙mL)	5079	−45.1	−62.1	10323	−44.9	−61.4
PE (mmHg∙mL)	1569	−23.5	−26.8	5376	−31.6	−34.7

**Table 5:** Impact of increases in heart rate and decreases in C_A_ on parameters of contractile state and ME.

Aortic Compliance	Normal			Stiff		
HR	60	100	140	60	100	140
		% Change from 60 bpm			% Change from 60 bpm	
ESPVR	1.37	−12.3	−13.2	1.43	17.8	29.9
dP/dt_max_–EDV	7.25	39.1	86.7	6.92	14.0	39.8
PRSW	82.5	−21.9	−30.7	119.9	−26.7	−39.5
EDV–PVA	100.6	−19.4	−21.6	182.1	−22.5	−28.0

ESPVR, end-systolic pressure–volume (slope); dP/dt_max_–EDV, dP/dt_max_–EDV (slope); PRSW, preload-recruitable SW slope; EDV–PVA, pressure–volume area–end-diastolic volume slope; ME, mechanical efficiency (%).

**Table 6:** Impact of increases in heart rate and decreases in C_A_ on eMVO_2_ and MyoEff.

Aortic Compliance	Normal			Stiff		
HR	60	100	140	60	100	140
		% Change from 60 bpm			% Change from 60 bpm	
eMVO_2_	9.3	19.5	43.7	14.2	18.7	47.4
MyoEff–SW (%)	22.5	−54.0	−73.8	29.9	−53.6	−73.8
MyoEff–PVA (%)	29.4	−49.8	−67.8	45.5	−49.8	−67.6
ME (%)	76.4	−8.5	−18.0	65.8	−7.6	−19.1

## Data Availability

The original contributions presented in the study are included in the article. Further inquiries can be directed to the corresponding author.

## Abbreviations

C_A_	Aortic compliance
CM	Computational model
eMVO_2_	Estimated myocardial oxygen consumption
MVO_2_	Myocardial oxygen consumption
MyoEff	Myocardial efficiency
MyoEff–PVA	Myocardial efficiency (PVA)
MyoEff–SW	Myocardial efficiency (SW)
PVA	Pressure–volume area
SW	Stroke work
VVC	Ventricular–vascular coupling
